# Reduced expression of somatostatin in GABAergic interneurons derived from induced pluripotent stem cells of patients with *parkin* mutations

**DOI:** 10.1186/s13041-019-0426-7

**Published:** 2019-01-18

**Authors:** Chizuru Iwasawa, Naoko Kuzumaki, Yukari Suda, Reiko Kagawa, Yuko Oka, Nobutaka Hattori, Hideyuki Okano, Minoru Narita

**Affiliations:** 1 Department of Pharmacology, Hoshi University School of Pharmacy and Pharmaceutical Sciences, Ebara, Shinagawa-ku, Tokyo, 142-8501 Japan; 2 Department of Physiology, Keio University School of Medicine, 35 Shinanomachi, Shinjuku-ku, Tokyo, 160-8582 Japan; 3 Life Science Tokyo Advanced Research Center (L-StaR), Hoshi University School of Pharmacy and Pharmaceutical Sciences, Ebara, Shinagawa-ku, Tokyo, 142-8501 Japan; 4 Department of Neurology, Juntendo University School of Medicine, Bunkyo, Tokyo, 113-8421 Japan

**Keywords:** Parkinson’s disease, iPSCs, GABA, Somatostatin

## Abstract

Parkinson’s disease (PD) is associated with both motor and non-motor symptoms, including constipation, sensory neuropathy, depression, dementia and sleep disorder. Somatostatin (SST) is considered to be a modulator of GABAergic inhibitory transmission, and its levels are reduced in cerebrospinal fluid of PD patients. In the present study, we evaluated the changes in the expression of SST in GABAergic neurons derived from induced pluripotent stem cells (iPSCs) of PD patients. Neural cells were co-treated with the Wnt antagonist IWP-2 and Shh during neurosphere formation to induce GABA-positive forebrain interneurons. Quantitative analyses showed no significant differences, but slight decreases, in the potency of differentiation into GABAergic neurons derived from iPSCs between healthy control and patients with *PARK2* mutations, who have been classified as a type of early-onset familial PD due to mutations in the *parkin* gene. Under this condition, the mRNA level of SST in GABAergic interneurons derived from iPSCs of PARK2-specific PD patients significantly decreased as neural maturation progressed. We also found that SST-positive GABAergic neurons were clearly reduced in GABAergic neurons derived from iPSCs of patients with *PARK2* mutations. These findings suggest that the reduction in the expression level of SST in GABAergic interneurons of PD may, at least partly, lead to complex PD-induced symptoms.

**Electronic supplementary material:**

The online version of this article (10.1186/s13041-019-0426-7) contains supplementary material, which is available to authorized users.

## Introduction

Parkinson’s disease (PD) is a progressive neurodegenerative disorder that afflicts about 4,000,000 patients worldwide [1]. While PD symptoms are mainly due to the progressive degeneration of neuronal cells in the substantia nigra [2], various other types of neural cells in the central and peripheral autonomic nervous systems also contribute to PD. Although a clinical diagnosis relies on the presence of motor symptoms, including akinesia, rigidity, resting tremor, and postural and balance difficulties, PD is also frequently associated with various non-motor symptoms, such as cognitive dysfunction, mood and psychotic disorder, which negatively affect the patient’s quality of life [3].

In the central nervous system (CNS), somatostatin (SST) is highly concentrated in a large proportion of GABAergic neurons, where it acts as a co-neurotransmitter or neuromodulator to modulate excitability and neuronal responses [4, 5]. SST has also been recognized as a marker for a particular GABAergic interneuronal subpopulation [6]. The level of SST in cerebrospinal fluid is significantly reduced in PD [7–10]. However, it has not yet been proven whether GABAergic neurons of PD patients show altered levels of SST.

A potential solution to the difficulty of modeling PD is to use reprogramming technology to generate disease-specific induced pluripotent stem cells (iPSCs). Recently, a method for controlling the regional identity of iPSC-derived neurons along the anteroposterior (A-P) and dorsoventral (D-V) axes was established [11]. In the present study, we evaluated possible changes in the expression level of SST in GABAergic neurons derived from PARK2-specific iPSCs.

## Methods

### Cell culture and neural differentiation

As controls, we used two human iPS cell lines: 201B7 iPSCs purchased from RIKEN BRC and were kindly provided by Dr. Shinya Yamanaka of Kyoto University [12] and WD39 iPSCs were established by Dr. Yoichi Imaizumi at Keio University. For PARK2 lines, patient A (PA9 and PA22) (female with an exon 2–4 deletion mutation) and patient B (PB2 and PB20) (male with an exon 6–7 deletion mutation) iPSCs were established previously [13]. All of the iPSCs were maintained on feeder cells in iPSC culture media, as described previously [13]. All of the experimental procedures for cell differentiation and analysis were approved by the respective Ethics Committees of Keio University School of Medicine (Approval Number: 20080016) and Hoshi University School of Medicine (Approval Number: 30–006). We applied a previously reported neural differentiation protocol [11, 14] to obtain forebrain GABAergic neurons from human iPSCs. For forebrain GABAergic neural induction, we added a Wnt inhibitor (2 μM IWP-2, FUJIFILM Wako Pure Chemical Corporation, Osaka, Japan) to the culture medium of NPCs from day 0 to day 13 and sonic hedgehog (100 ng/ml Shh, R&D Systems Inc., Minneapolis, MN, USA) and a Shh agonist (1 μM Purmorphamine, Calbiochem, San Diego, CA, USA) to the culture medium of neural progenitor cells (NPCs) from days 2 to 13. Subsequently, NPCs on day 13 were dissociated into single cells using TrypLE™ Select (Thermo Fisher Scientific, Waltham, MA, USA) and seeded on culture dishes coated with poly-L-ornithine (Sigma-Aldrich, St. Louis, MO, USA) and fibronectin (Sigma-Aldrich). For neural differentiation, cells were cultured in KBM Neural Stem Cell medium (KOHJINBIO) containing B27 (Thermo Fisher Scientific), 20 ng/mL brain-derived neurotrophic factor (BDNF, R&D Systems), 20 ng/mL glial cell-derived neurotrophic factor (GDNF, R&D Systems), 200 μM ascorbic acid (Sigma-Aldrich), and 500 μM dibutyryl-cAMP (Sigma-Aldrich).

### Quantitative real-time PCR

Total RNA was extracted from control and PARK2-derived cells for each developmental stage, iPSCs, NSCs and differentiated neurons, using TRIzol reagent (Thermo Fisher Scientific) and an RNeasy mini Kit (QIAGEN, Venlo, The Netherlands). Complementary DNA was synthesized using a SuperScript® VILO™ cDNA Synthesis Kit (Thermo Fisher Scientific). Gene expression levels were measured by quantitative real-time PCR (qPCR) on a StepOnePlus™ Real-Time PCR System with a 96-well format. Primer sequences are listed in Additional file 1: Table S1: Quantitative PCR was performed with 5 ng cDNA per well in a 10 μl reaction using Fast SYBR® Green Master Mix (Thermo Fisher Scientific). The data were assessed using the ΔΔ Ct method and normalized by β-actin expression.

### Immunocytochemistry

Differentiated neurons were fixed with 4% paraformaldehyde and 0.1 M phosphate buffer for 20 min at room temperature. After being washed with PBS, samples were blocked with 5% BSA and 0.3% Triton X-100 in PBS for 1 h. Primary antibodies were diluted in 1% BSA and 0.3% Triton X-100 in PBS and applied at 4 °C overnight. This was followed by incubation with appropriate secondary antibodies (Thermo Fisher Scientific) conjugated with AlexaFluor 488, AlexaFluor 546/594 or AlexaFluor 647 (1:1000) for 90 min at room temperature. Samples were mounted with DAPI-Fluoromount-G™ (SouthernBiotech, Birmingham, AL, USA) and immunoreactive cells were visualized using a BZ-X710 (KEYENCE, Osaka, Japan). The primary antibodies used were as follows: anti-β-III tubulin (1:1000, Sigma-Aldrich), anti-GABA (1:1000, Sigma-Aldrich), anti-SST (1:500, Millipore), Anti-Parvalbumin (PV; 1:2000, Sigma-Aldrich).

### Statistical analysis

All data are presented as the mean ± standard error of mean (SEM). The statistical significance of differences was analyzed by GraphPad Prism (GraphPad Software, La Jolla, CA, USA). For the comparison of multiple groups, statistical analysis was performed using one-way analysis of variance (ANOVA) with Bonferroni’s post-hoc test. Probability values less than 0.05 (*p* < 0.05) were considered to be statistically significant.

## Results

### Differentiation of human iPSCs to GABAergic neurons

**Fig. 1:**
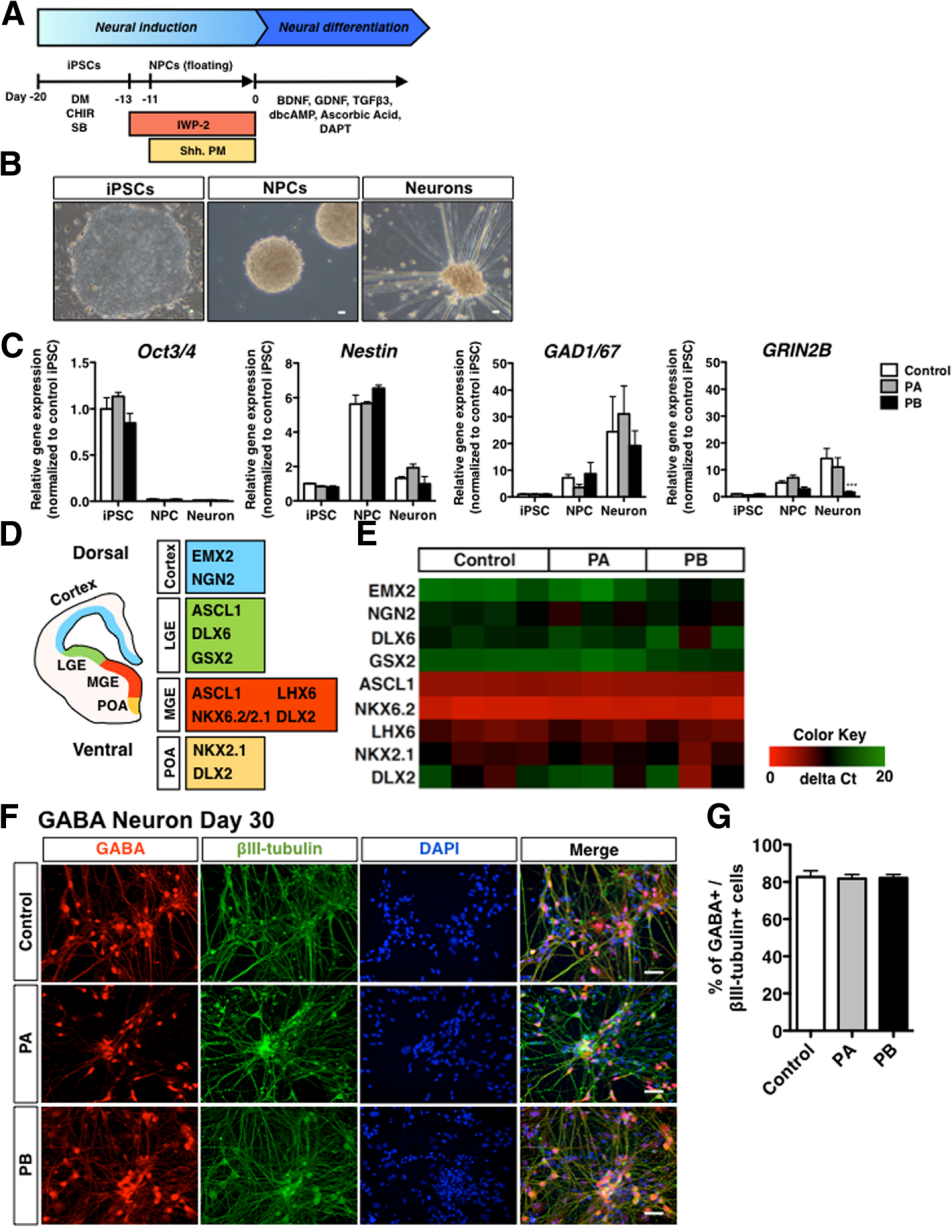
Differentiation of human iPSCs to GABAergic neurons. (**a**) Neural induction and differentiation protocol. Shh, sonic hedgehog; PM, purmorphamine. (**b**) Phase-contrast images of iPSCs, neural progenitor cells (NPCs) and GABAergic neurons (day 30) derived from control. Scale bar, 50 μm (**c**) Quantitative gene-expression analysis of each neurodevelopmental stage marker (Oct3/4, pluripotent stem cell marker; Nestin, neural stem cell marker; GAD1/67, GABAergic neuron marker) using iPSCs, NPCs and GABAergic neurons (day 30) derived from control and PARK2 (PA and PB). Control is presented as one column combining and averaging two cell lines (WD39 and 201B7). Data are presented as means ± SEM. Statistical analysis was performed using one-way ANOVA with Bonferroni’s post-hoc test (*n* = 3–4), ***p < 0.001 compared to control. (**d**) Schematic representation of the dorsal-ventral markers in the telencephalon. LGE, lateral ganglionic eminence; MGE; medial ganglionic eminence; POA, preoptic area. (**e**) Quantitative gene-expression analysis of control- and PARK2 (PA and PB)-derived NPCs for the expression of dorsal-ventral markers in the telencephalon. (**f**) Immunocytochemical analysis for the GABAergic neuron marker GABA and the neuronal marker βIII-tubulin of control and PARK2 (PA and PB) iPSC-derived GABAergic neurons. Scale bar, 50 μm. (**g**) Quantification of the percentages of βIII-tubulin- and GABA-positive cells. Data are presented as means ± SEM. Statistical analysis was performed using one-way ANOVA with Bonferroni’s post-hoc test (*n* = 3)

It has been demonstrated that the A-P identity of iPSC-derived neural progenitors can be controlled by Wnt signaling during neurosphere formation [11]. We applied this previously reported method to generate a GABAergic neuron-enriched culture by treating iPSCs-derived cells with the Porcupine inhibitor IWP-2 [15], which leads to the formation of forebrain cells, including GABAergic neurons [11, 16]. In addition, Shh activation converted the D-V identity from dorsal to ventral without perturbing the A-P identity [11]. Since GABAergic neurons originate from the medial ganglionic eminence (MGE), we treated cells with the Shh protein and purmorphamine during neurosphere formation (Fig. 1a, b). When neurotrophic factors including BDNF were used for neural maturation, NPCs differentiated into GABAergic neurons (Fig. 1a, b). We next investigated the expression of cell-type-specific markers during differentiation from iPSCs. We confirmed that iPSCs expressed an undifferentiated cell-marker, Oct3/4, while NPCs expressed the neural stem cell marker Nestin (Fig. 1c). Moreover, the GABA-producing enzyme, glutamate decarboxylase (GAD1/67) gradually increased as cell differentiation progressed (Fig. 1c). There were no significant differences of the expression levels of Oct3/4, Nestin and GAD1/67 between control and PD (PA and PB) lines (Fig. 1c). Although we observed a slight increase in the mRNA level of GRIN2B, which is an excitatory neuronal marker, in Day 30 culture neurons compared to that in iPSCs (Fig. 1c, ***p < 0.001 vs. control), GAD1/67 expression was observed with higher levels in control and PARK2 (PA and PB) iPSCs-derived neurons. We also observed the expression of telencephalon-specific D-V markers. The MGE markers ASCL1 and NKX6.2 were highly expressed in NPCs derived from control and PARK2 (PA and PB)-specific iPSCs (Fig. 1e). After 30 days of neural maturation, the differentiated cells were labeled by antibodies to βIII-tubulin (a neuron-specific marker) and GABA (GABAergic neuronal marker) (Fig. 1f). About 80% of βIII-tubulin-positive neurons expressed GABA, indicating successful GABAergic neuronal differentiation (Fig. 1g). Quantitative analyses showed no differences in the potency of differentiation into GABAergic neurons derived from iPSCs between healthy control and PARK2 patients at 30 days (Fig. 1g).

### Characterization of GABAergic neurons derived from PARK2-specific iPSCs

**Fig. 2:**
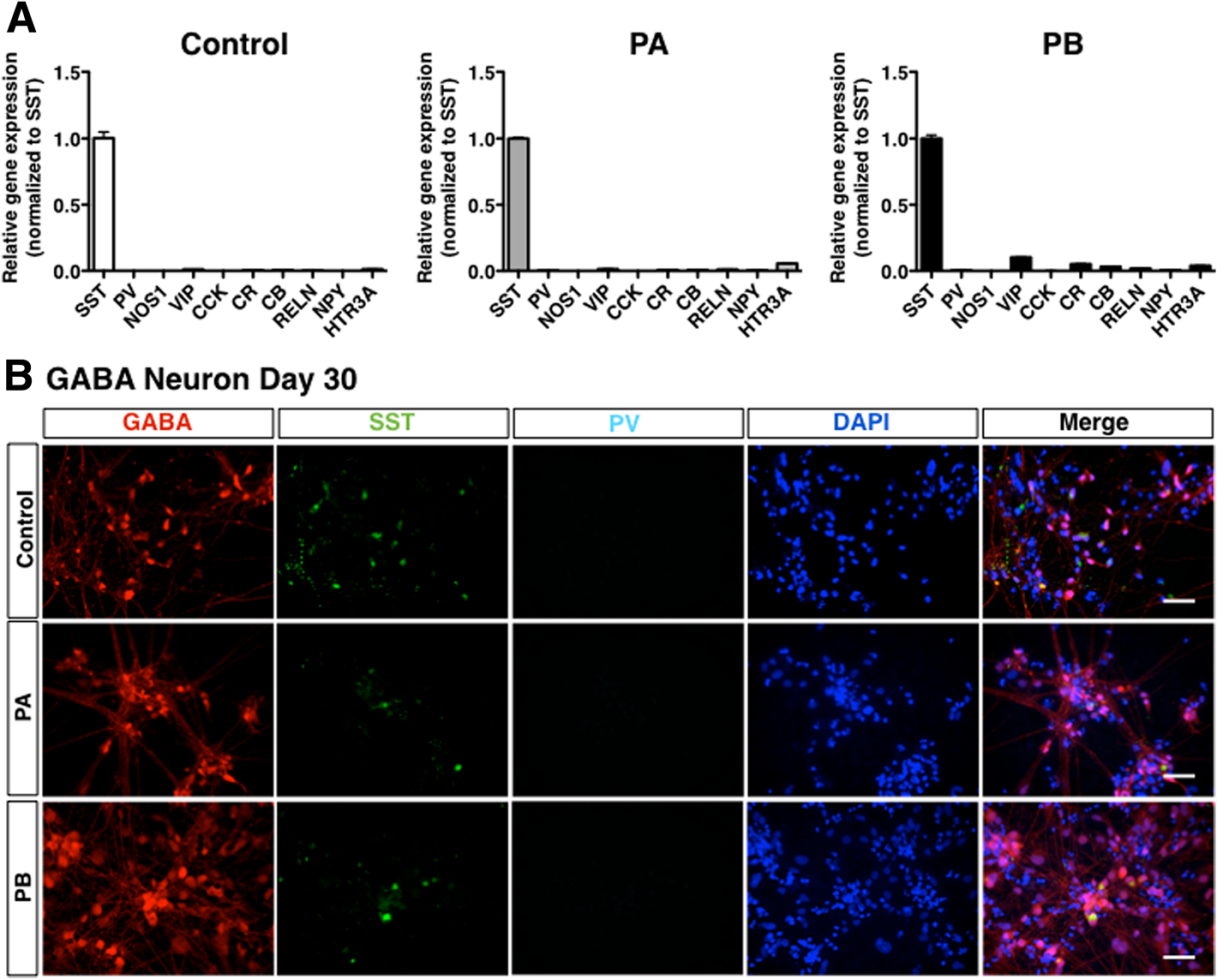
Identification of subtypes of GABAergic neurons derived from human iPSCs. (**a**) Quantitative gene-expression analysis of the subtypes of GABAergic neurons revealed that the main population of GABAergic neurons was SST-expressing neurons in control-and PARK2 (PA and PB)-derived GABAergic neurons (day 30, *n* = 3). Abbreviations; SST, somatostatin; PV, parvalbumin; NOS1, nitric oxide synthase 1; VIP, vasoactive intestinal peptide; CCK, cholecystokinin; CR, calretinin; CB, calbindin; RELN, reelin; NPY, neuropeptide Y; HTR3A, 5-hydroxytryptamine receptor 3A. (**b**) Immunocytochemical analysis for the major subtypes of GABAergic interneuron markers, SST and PV, and the GABAergic neuron marker (GABA) of PARK2 (PA and PB) and control iPSC-derived GABAergic neurons. Scale bar, 50 μm

Cortical interneurons are conventionally categorized based on neuropeptide and Ca^2+^ binding protein expression [17]. To characterize GABAergic neurons, we investigated the expression of GABAergic interneuron-specific markers in differentiated cells derived from control and PARK2 (PA and PB)-specific iPSCs. We found that only SST was highly expressed, while the expression of other markers, including PV, nitric oxide synthase (NOS) 1, vasoactive intestinal polypeptide (VIP), cholecystokinin (CCK), calretinin (CR), calbindin (CB), reelin (RELN), neuropeptide Y (NPY) and 5-hydroxytryptamine receptor 3A (HTR3A), was very low in differentiated GABAergic neurons (Fig. 2a). Although SST-positive GABAergic neurons were detected at different levels in differentiated cells derived from control and PARK2 (PA and PB)-specific iPSCs, no PV-positive GABAergic neurons were observed in differentiated cells derived from iPSCs from either source (Fig. 2b). These results suggest that forebrain neurons derived from control and PARK2-specific iPSCs resemble GABAergic interneurons.

### Decreased expression of SST in PARK2-specific iPSCs-derived GABAergic neurons

**Fig. 3:**
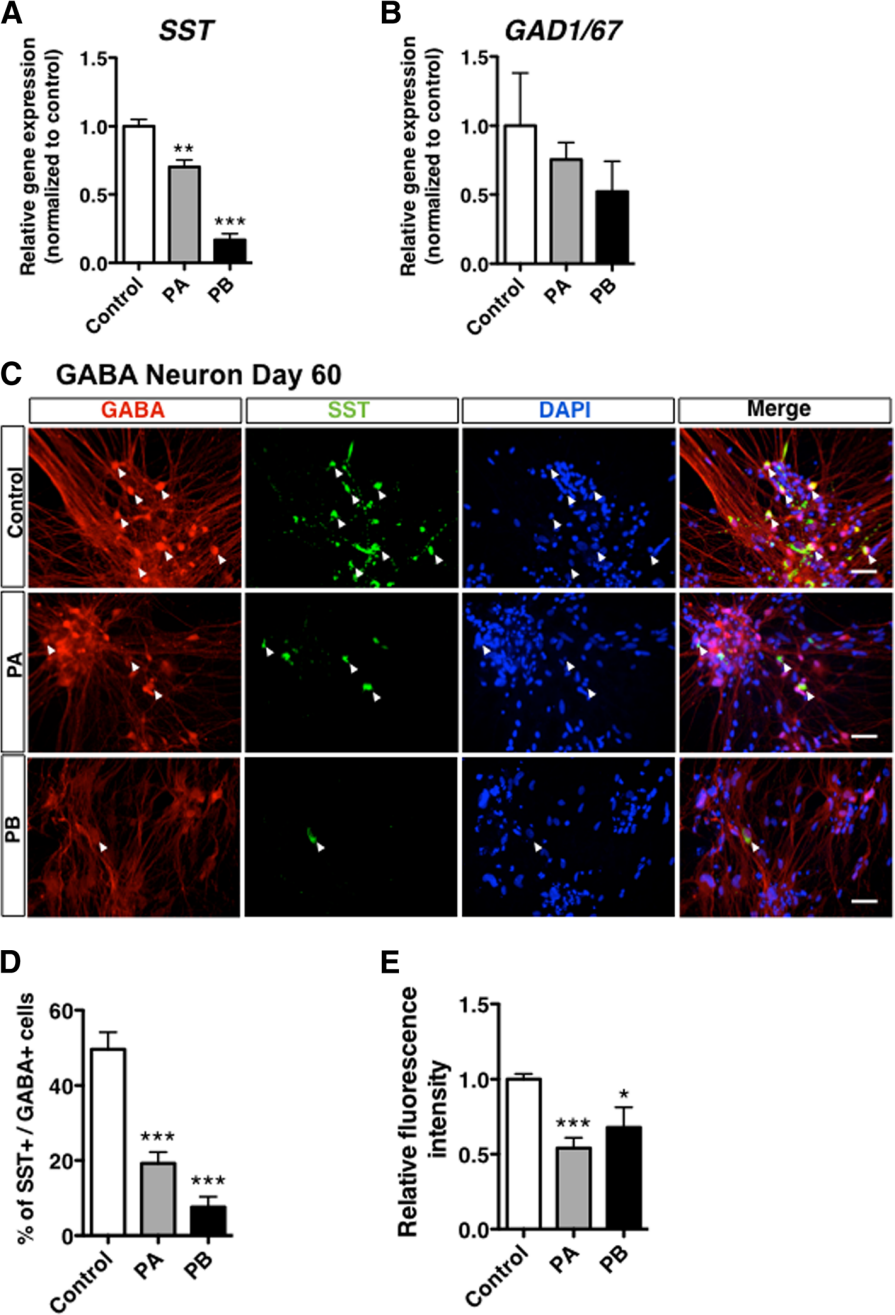
Changes in the expression of SST in PARK2-derived GABAergic neurons. (**a**) Quantitative gene-expression analysis of SST in control and PARK2 (PA and PB) iPSC-derived GABAergic neurons (day 60). Control is presented as one column combining and averaging two cell lines (WD39 and 201B7). Data are shown as means ± SEM. Statistical analysis was performed using one-way ANOVA with Bonferroni’s post-hoc test (*n* = 5), **p < 0.01, ***p < 0.001 compared to control. (**b**) Quantitative gene-expression analysis of GAD1/67 in control and PARK2 (PA and PB) iPSC-derived GABAergic neurons (day 60). Control is presented as one column combining and averaging two cell lines (WD39 and 201B7). Data are shown as means ± SEM. Statistical analysis was performed using one-way ANOVA with Bonferroni’s post-hoc test (n = 5). (**c**) Immunocytochemical analysis for GABA and SST of control and PARK2 (PA and PB) iPSC-derived GABAergic neurons (day 60). Scale bar, 50 μm. (**d**) Quantification of the percentages of SST- and GABA-positive cells derived from control and PARK2 (PA and PB) iPSCs. Data are presented as means ± SEM. Statistical analysis was performed using one-way ANOVA with Bonferroni’s post-hoc test (n = 3), ***p < 0.001 compared to control. (**e**) Quantification of fluorescence intensity for SST levels. Data are presented as means ± SEM. Statistical analysis was performed using one-way ANOVA with Bonferroni’s post-hoc test (*n* = 3), *p < 0.05, ***p < 0.001 compared to control

We next investigated possible changes in the expression of SST at 60 days after neural differentiation from control and PARK2-specific iPSCs. We observed a significant decrease in mRNA levels of SST in PARK2 (PA and PB)-specific iPSCs-derived GABAergic neurons at 60 days of differentiation (Fig. 3a, **p < 0.01, ***p < 0.001 vs. control). In GABAergic neurons derived from PARK2-specific iPSCs, the number of SST-positive GABAergic neurons was significantly reduced (Fig. 3c, d), which was also confirmed by the reduction in fluorescence intensity of SST (Fig. 3e). Under these conditions, no significant differences, but slight decreases, in mRNA levels of GAD1/67 in GABAergic neurons were found between healthy control and PARK2 (PA and PB) patients (Fig. 3e). In addition, we found the tendency of the reduction in SST expression normalized with GAD1/67 expression (Additional file 2: Figure S1).

## Discussion

PD is a neurodegenerative disease that is characterized by motor dysfunctions such as tremor, slowness, stiffness, and balance problems. These symptoms are associated with a progressive loss of dopaminergic (DAnergic) neurons in the substantia nigra. However, other classical neurotransmitters such as acetylcholine, glutamate and GABA also play a crucial role in PD-related motor coordination [18–20]. On the other hand, PD patients also have non-motor symptoms which are undoubtedly accompanied by a deficit of GABA [21, 22]. In our previous study, we successfully generated DAnergic neuron-enriched culture by treatment with shh, CHIR and purmorphamine during neurosphere formation [14]. By using these cells, we demonstrated that an iPSCs-based model of PARK2 recapitulated the vulnerability of DAnergic neurons with a significant increase in ROS production [23, 24] and ghrelin receptor dysfunction [25]. Recent reports have suggested that the A-P identity, ranging from the telencephalon to the spinal cord, can be controlled by treatment with three factors, IWP-2, CHIR, and RA, during neurosphere formation [11]. In addition to DAnergic neurons, we also generated GABAergic interneuron-enriched culture by treatment of PARK2-specific iPSCs-derived cells with IWP-2, Shh protein and purmorphamine [11]. The molecular composition of GABAergic neurons was diverse, and included the expression of calcium-binding proteins PV, CR and CB, and neuropeptides, such as SST, NPY, CCK and VIP [26]. In recent studies, different subgroups of GABAergic interneurons, including CR, CB, CCK, PV, SST and NPY, have been identified by immunohistochemistry and systematically counted in the anterior cingulate, prelimbic and infralimbic cortices [27]. To characterize the present GABAergic neurons derived from iPSCs, we investigated the expression of subtype-specific markers. As a result, only SST was highly expressed, while other markers (PV, NOS1, VIP, CCK, CR, CB, RELN, NPY and HTR3A) were seen at very low levels.

SST, which inhibits several biologically active substances such as growth hormone (GH) and insulin [28, 29], is abundant throughout the CNS [30]. SST acts as a neurotransmitter (co-transmitter) or neuromodulator and plays a role in the fine-tuning of neuronal activity and synaptic plasticity. The changes in GABAergic function in neural microcircuits could result in modulation of the excitatory-inhibitory imbalance via the release of SST. It has been considered that the decreased SST levels in the frontal and entorhinal cortex as well as the hippocampus lead to the cognitive deficits in patients with PD [9]. Furthermore, the expression levels of CB, SST and CCK, which are markers of inhibitory neuronal subpopulations in GABAergic interneurons of the frontal cortex, are known to be negatively correlated with age [31]. In the present study, we found that the mRNA level of SST and the number of SST-positive GABAergic neurons were clearly decreased in GABAergic interneurons derived from PARK2 patient-specific iPSCs. In the previous study, PARK2 mutations lost the ability to suppress MAO transcription and resulted in elevation of oxidative stress in PARK2 iPS-derived DAnergic neurons [32]. A recent study suggested that SST exerted neuroprotective effects against the lipopolysaccharide (LPS)-mediated loss of TH-positive DA neurons in the substantia nigra of a PD model [33]. Although further studies are needed to investigate the role of SST in the dysfunction of the neural network in PD, we hypothesize that PARK2 mutations somehow could affect the gene transcription and mitochondrial dysfunction in SST-expressing GABAergic neurons.

In conclusion, the present study clearly indicated that the level of SST was reduced in differentiated GABAergic interneurons from PARK2-specific iPSCs derived from PD patients. We propose that the deficiency of SST in GABAergic neurons of the frontal cortex may lead to the motor and/or non-motor symptoms of PD via the regulation of the excitatory-inhibitory imbalance of the neural network.

## Additional files

Additional file 1: **Table S1.** Real-time PCR primer. (PDF 99.6 kb)

Additional file 2: **Figure S1.** Expression of SST in GABAergic neurons normalized with GAD1/67 expression. Quantitative gene-expression analysis of SST in control and PARK2 (PA and PB) iPSC-derived GABAergic neurons (day 60) normalized with GAD1/67 expression. (TIFF 97.3 kb)

## Abbreviations

BDNF: Brain-derived neurotrophic factor
BSA: Bovine serum albumin
CB: Calbindin
CCK: Cholecystokinin
CHIR: CHIR99021
CNS: Central nervous system
CR: Calretinin
FBS: Fetal bovine serum
GABA: Gamma amino butyric acid
GAD1/67: Glutamate decarboxylase 1/67
GDNF: Glial cell-derived neurotrophic factor
GH: Growth hormone
HTR3A: 5-hydroxytryptamine receptor 3A
iPSC: Induced pluripotent stem cell
LPS: Lipopolysaccharide
NOS: Nitric oxide synthase
NPC: Neural progenitor cell
NPY: Neuropeptide Y
PD: Parkinson’s disease
PFA: Paraformaldehyde
PV: Parvalbmin
RELN: Reelin
Shh: Sonic hedgehog
SST: Somatostatin
VIP: Vasoactive intestinal polypeptide
