# Supplementary material for: Reduced expression of somatostatin in GABAergic interneurons derived from induced pluripotent stem cells of patients with parkin mutations
Source: Mol Brain. 2019 Jan 18;12:5. doi: 10.1186/s13041-019-0426-7 (PMC6339354; doi:10.1186/s13041-019-0426-7)
Supplement: Supplementary file 1 — Table S1. Real-time PCR primer. (PDF 99.6 kb) [file 13041_2019_426_MOESM1_ESM.pdf]

**Table S1. Real-time PCR primer**

| Symbol | Forward primer (5'→3') | Reverse primer (5'→3') |
|---|---|---|
| ASCL1 | GATGAGTAAGGTGGAGACACTGCG | CCGACGAGTAGGATGAGACCG |
| CB | TGGATCAGTATGGGCAAAGAGA | ATCGGAAGAGCAGCAGGAAAT |
| CCK | AGGGTATCGCAGAGAACGGA | CTTATCCTGTGGCTGGGGTC |
| CR | TGGAAGCACTTTGACGCAGAC | CAGAGCCTTTCCTTGCCTTCT |
| DLX2 | ACGCTCCCTATGGAACCAGTT | TCCGAATTTCAGGCTCAAGGT |
| DLX6 | CCGAACTGGCAGCTTCCTTAG | GGGTCGCTCTCATGAGGATTAC |
| EMX2 | GCTTCTAAGGCTGGAACACG | CCAGCTTCTGCCTTTTGAAC |
| GAD1/67 | GCCAGACAAGCAGTATGATGT | CCAGTTCCAGGCATTTGTTGAT |
| GSX2 | GAGATTCCACTGCCTCACCATG | CTCCGGAGTCGAGACAGGTAC |
| HTR3A | GGGTGCTGCCCTACTTTCG | GAAGATGCTGGGCAGTAGCAG |
| LHX6 | GCAGAACAGCTGCTACATCAAGAA | CAGTCGCTGGCGTAGATCTGTC |
| Nestin | TTCCCTCAGCTTTCAGGACCCCAA | CAGTCGCTGGCGTAGATCTGTC |
| NGN2 | TGGGTCTGGTACACGATTGC | GTCTTCTTGATGCGCTGCAC |
| NKX2.1 | GCCGTACCAGGACACCATGA | GCTCATGTTCATGCCGCTCG |
| NKX6.2 | TCCTGGACAAGGACGGGAAG | ACTTGGTCTGCTCGAAGGTTT |
| NOS1 | CCTATGGGGGAAGGGCAATG | TTGACCTTGAGGAAGCGTGG |
| NPY | TGTTCCCAGAACTCGGCTTG | TGCATTGGTAGGATGGGTGG |
| OCT3/4 | AAGCGAACCAGTATCGAGAACC | CTGATCTGCTGCAGTGTGGGT |
| PV | TGACAGACTTGCTGAACGCT | TCAGGCCGACCATTTGGAAG |
| SST | CCCCAGACTCCGTCAGTTTCT | CATTCTCCGTCTGGTTGGGT |
| RELN | GCTTGCTGGTGACAGGACTA | GAGAGGCTACCACACTGCAC |
| VIP | TCTCACAGACTTCGGCATGG | TCATTTGCTCCCTCAAAGGGT |
| β-actin | GATCAAGATCATTGCTCCTCCT | GGGTGTAACGCAACTAAGTCA |
