# Supplementary figures and images for: Reduced expression of somatostatin in GABAergic interneurons derived from induced pluripotent stem cells of patients with parkin mutations
Source: Mol Brain. 2019 Jan 18;12:5. doi: 10.1186/s13041-019-0426-7 (PMC6339354; doi:10.1186/s13041-019-0426-7)

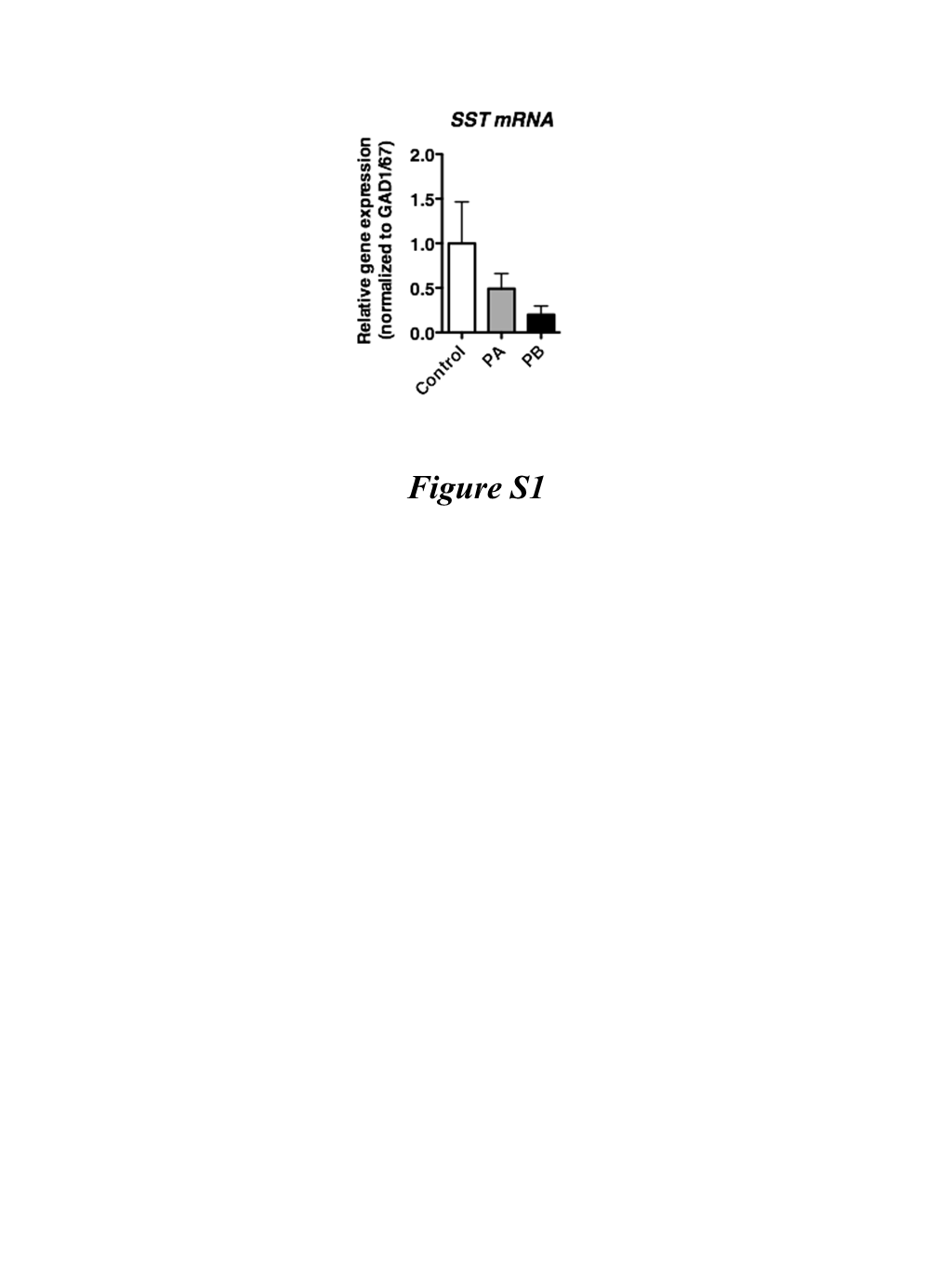

Supplement: Supplementary file 2 — Figure S1. Expression of SST in GABAergic neurons normalized with GAD1/67 expression. Quantitative gene-expression analysis of SST in control and PARK2 (PA and PB) iPSC-derived GABAergic neurons (day 60) normalized with GAD1/67 expression. (TIFF 97.3 kb) [file 13041_2019_426_MOESM2_ESM.tiff]
